# Editorial for the Special Issue on Micro-Electro Discharge Machining: Principles, Recent Advancements and Applications, Volume II

**DOI:** 10.3390/mi14010029

**Published:** 2022-12-23

**Authors:** Irene Fassi, Francesco Modica

**Affiliations:** 1 STIIMA CNR, Institute of Intelligent Industrial Technologies and Systems for Advanced Manufacturing, National Research Council, Via A. Corti 12, 20133 Milan, Italy; 2 STIIMA CNR, Institute of Intelligent Industrial Technologies and Systems for Advanced Manufacturing, National Research Council, Via P. Lembo 38/F, 70124 Bari, Italy

The second volume of the Special Issue on “Micro-Electro Discharge Machining: Principles, Recent Advancements and Applications” confirms the growing interest in the micro-EDM technology as a suitable and efficient technology for machining novel, multi-material components, with demanding requirements in terms of precision, accuracy and productivity.

This volume consists of 10 original research papers which involve several approaches to micro-EDM and cover the enhancement of the process performance, such as the material removal rate, surface roughness, or accuracy of the machining, using advanced optimization methods. Some studies also consider several dielectric fluid additives and investigate the processability of new materials. Others investigate the combination of Reverse-micro-EDM with laser beam micromachining or explore new applications for the micro-EDM for fabricating antimicrobial nanosilver colloid.

In more detail, in order to improve the machining accuracy of detail features in micro-EDM milling, a theoretical model is developed by Jing et al. [1] to simulate the micro-EDM milling process with a straight-line single path. In particular, the model is obtained by the accumulative difference mechanism in time and space. Micro-EDM milling experiments were carried out to verify the simulation model, showing that the maximum mean relative deviation between the microgroove profiles of simulation and experiments is 11.09%, with a good consistency in profile shapes.

The influence of the near-dry WEDM technique to reduce the environmental impact of wet WEDM was investigated by Chaudhari et al. [2]. The study employed a teaching–learning-based optimization (TLBO) algorithm to find the optimal combination of process parameters for material removal rate (MRR) and surface roughness (SR) considering near-dry WEDM of NiTiNol Shape Memory Alloy. Even if near-dry WEDM shows lower MRR in respect of wet-WEDM, it can machine a better surface morphology in terms of reduction in surface defects and better surface quality.

A mathematical–statistical computational (MSC) model for predicting high productivity and quality of the machined area is formulated by Straka et al. [3] by the application of non-linear programming (NLP) methods using MATLAB. The method is applied to maximize the process performance of micro-WEDM on a workpiece made of steel MS1 sintered via direct metal laser sintering (DMLS). Experimentation and results show the model’s effectiveness in optimizing process performance.

Wire-cut electro-discharge machining (Wire-EDM) of polymer composite material (PCM) was studied by Ablyaz et al. [4]. Tests were performed on a workpiece made of a laminated fibrous polymer composite with carbon fiber twill as reinforcement/filler and epoxy as a binder material. The machining can be performed thanks to improved conductivity obtained using 1 mm thick titanium plates sandwiched on the PCM. The results demonstrated that voltage and pulse duration and their interaction are the significant factors affecting the cut-width accuracy for machining the PCM workpiece.

Sidhu et al. [5] used the analytic hierarchy process (AHP), a multiple-criteria decision-making technique, to achieve a reliable outcome for different responses in electric discharge machining (EDM) of metal matrix composites (MMCs). They identified the optimal process conditions by considering two materials, 65 vol% SiC/A356.2, and 10 vol% SiC-5 vol% quartz/Al composites, revealing that, in the presence of a suspended particle dielectric medium (PMEDM), a graphite tool electrode and higher pulse-on time coupled with lowest pulse-off time contributed to minimizing the residual stress with the desired MRR.

Ablyaz et al. [6] investigated a hybrid magnetic field assisted powder mixed electrical discharge machining on machining the aluminum-silicon carbide (Al-SiC) metal matrix composite to obtain a higher surface finish and enhanced the material removal rate. The dielectric mediums employ plain EDM oil, SiCp mixed, and graphite powder mixed EDM oil. They find that MRR augmented considerably with increased magnetic field intensity and peak current. At the same time, the quality of the machined surface improved significantly in graphite powder mixed dielectric flushing conditions with an intermediate external magnetic field environment. Moreover, micro-hardness enhancement was quantified as compared to base material due to the transfer of the material (SiCp).

Gattu et al. [7] mixed three different powders at different concentrations in a dielectric fluid: electrically conductive carbon nanofiber (CnF), semiconductive silicon (Si) powder, and insulative alumina powder (Al_2_O_3_). The study evaluated effects on material removal rate (MRR), relative electrode wear rate (REWR), and surface roughness on machining (EDM) of ultrafine particle type tungsten carbide and observing single discharge crater and hole machining tests. The results showed that adding CnF enhanced the material removal rate under all conditions. In contrast, Si and Al_2_O_3_ powders only improved the machining performance at a high discharge energy of 110 V. Improvement in surface roughness was observed prominently at high voltages for all the powders. However, alumina improved the surface roughness the most among the three powders.

Esser et al. [8] observed the discharge phenomena in the discharge gap by using a high-speed camera to study the effect on the machining process of tool vibration used to improve flushing conditions. They found that the discharges occurred in periodic intervals, and the intensity increased with the amplitude of tool vibration. Consequently, it was determined that, by adjusting the vibration parameters, it is possible to achieve optimum stability by improving the discharge distribution uniformity, increasing the machining efficiency and reducing the tool wear.

Reverse-µEDM was considered by Kishore et al. [9] with the fabrication of the tool plate realized by Nd:YAG-based laser beam micromachining (LB µM). The Grey relation analysis technique was used for optimizing LBµM parameters for producing tool plates with arrayed micro-holes in elliptical and droplet profiles. A duty cycle of 1.25% and a current of 20% were found to be an optimal setting for the fabrication of burr-free shallow striation microholes with a minimal dimensional error. After that, analogous protrusions were produced by Reverse-µEDM. Since the tool has no apparent cleavage or burrs at the micro-hole cut edges, it allows faster machining by restricting high-order discharges and short-circuiting during reverse-µEDM and obtaining protrusions free from tip damage.

Finally, an alternative study has been performed by Tseng et al. [10] with the implementation of a Micro-EDM Monitoring System to Fabricate Antimicrobial Nanosilver Colloid. The new system can replace the traditional oscillograph observation method, and its advantage consists of instantly observing and controlling discharge conditions. The monitoring system can use the discharge rate to control the energy consumption of the electrodes to standardize the nanosilver colloid. By experimentation, it was found that the nanosilver colloid prepared by EDM is free of any chemical additive that, compared to other preparation methods, is more applicable to biotechnology and the human body.

We thank all the authors who submitted their papers to this Special Issue, “Micro-Electro Discharge Machining: Principles, Recent Advancements and Applications”. We would also like to acknowledge all the reviewers for dedicating their time to providing careful and timely reviews to ensure the quality of this Special Issue.
